# PET Imaging of Dopamine Neurotransmission During EEG Neurofeedback

**DOI:** 10.3389/fphys.2020.590503

**Published:** 2021-01-11

**Authors:** Tomas Ros, Jessica Kwiek, Theo Andriot, Abele Michela, Patrik Vuilleumier, Valentina Garibotto, Nathalie Ginovart

**Affiliations:** ^1^Department of Basic Neurosciences, University of Geneva, Geneva, Switzerland; ^2^CIBM Center for Biomedical Imaging, Lausanne, Switzerland; ^3^Department of Psychiatry, University of Geneva, Geneva, Switzerland; ^4^Division of Nuclear Medicine and Molecular Imaging, Department of Medical Imaging, Geneva University Hospitals, Geneva, Switzerland

**Keywords:** dopamine, neurofeedback, positron emission tomography, electromyography, electroencephalography, Fallypride

## Abstract

Neurofeedback (NFB) is a brain-based training method that enables users to control their own cortical oscillations using real-time feedback from the electroencephalogram (EEG). Importantly, no investigations to date have directly explored the potential impact of NFB on the brain’s key neuromodulatory systems. Our study’s objective was to assess the capacity of NFB to induce dopamine release as revealed by positron emission tomography (PET). Thirty-two healthy volunteers were randomized to either EEG-neurofeedback (NFB) or EEG-electromyography (EMG), and scanned while performing self-regulation during a single session of dynamic PET brain imaging using the high affinity D_2/3_ receptor radiotracer, [^18^F]Fallypride. NFB and EMG groups down-regulated cortical alpha power and facial muscle tone, respectively. Task-induced effects on endogenous dopamine release were estimated in the frontal cortex, anterior cingulate cortex, and thalamus, using the linearized simplified reference region model (LSRRM), which accounts for time-dependent changes in radiotracer binding following task initiation. Contrary to our hypothesis of a differential effect for NFB vs. EMG training, significant dopamine release was observed in both training groups in the frontal and anterior cingulate cortex, but not in thalamus. Interestingly, a significant negative correlation was observed between dopamine release in frontal cortex and *pre-to-post* NFB change in spontaneous alpha power, suggesting that intra-individual changes in brain state (i.e., alpha power) could partly result from changes in neuromodulatory tone. Overall, our findings constitute the first direct investigation of neurofeedback’s effect on the endogenous release of a key neuromodulator, demonstrating its feasibility and paving the way for future studies using this methodology.

## Introduction

Cortical oscillations are generated by collective fluctuations of synaptic and somatic membrane potentials (Buzsáki et al., 2012), and therefore closely reflect excitability changes of neuronal populations (Rossini et al., 1991; Haegens et al., 2011; Schalk et al., 2017). Behavioural states of attention/vigilance have been consistently tied to dynamic decreases (also known as desynchronization) of low-frequency electroencephalogram (EEG) rhythms (i.e.,<15 Hz) (Harris and Thiele, 2011; Luczak et al., 2013; Mcginley et al., 2015; Zerlaut and Destexhe, 2017), that otherwise dominate the cortical activity during quiet “resting.” In waking adult humans, the dominant resting-state rhythm is the alpha (8–12 Hz) rhythm (Groppe et al., 2013). Alpha rhythm increases or decreases, respectively, has been found to reflect neural inhibition and excitation of sensory cortices (Romei et al., 2008; Haegens et al., 2011), acting as an inhibitory gate for external stimuli (Cooper et al., 2003; Jensen and Mazaheri, 2010; Luczak et al., 2013). On task, lapses of sensory detection (Ergenoglu et al., 2004; O’Connell et al., 2009), motor inhibition (Mazaheri et al., 2009), and subjective attention (Macdonald et al., 2011) have all been related to higher trial-by-trial levels of alpha synchronization.

Interestingly, at the synaptic level, cortical oscillatory activity is known to be neurochemically regulated by a complex cocktail of neurotransmitters/neuromodulators, including dopamine (Lee and Dan, 2012). Hence, studies in humans have shown that stimulation of dopaminergic pathways may concomitantly modify attention and resting-state EEG rhythms. In healthy adults for example, treatment with the indirect dopaminergic/noradrenergic agonist methylphenidate is able to improve target detection by significantly reducing alpha oscillations which preceded lapses of attention (Dockree et al., 2017). Likewise, methylphenidate significantly suppressed theta/alpha power in adults with ADHD who were classified as clinical responders (Bresnahan et al., 2006). Conversely, selective dopaminergic antagonists have been found to enhance alpha power and degrade cognitive performance in animals (Puig and Miller, 2015). Moreover, a simultaneous EEG and positron emission tomography (PET) study revealed endogenous striatal dopamine release to inversely correlate with power of alpha rhythms during meditation (Kjaer et al., 2002). Importantly, the aforementioned *in vivo* studies are compatible with *in vitro* evidence that dopaminergic agonists decrease low-frequency EEG rhythms (Sebban et al., 1999) while antagonists increase them (Sebban et al., 1999), and this has been directly linked to activation of dopamine receptors (Popoli et al., 1996; Chen et al., 2013).

These collective findings suggest there may be a common electrochemical mechanism linking the release of neuromodulators (such as dopamine) and the expression of low-frequency EEG rhythms (such as alpha oscillations). The cortex has strong reciprocal connections with the dopaminergic system and its subcortical nuclei. A major pathway involves dopamine neurons localized in the ventral tegmental area and projecting to the medial prefrontal cortex (Lohani et al., 2019) and the anterior cingulate cortex (ACC) (Steullet et al., 2014). In addition, the thalamus has also been shown to exhibit dopamine transmission during attentional states (Christian et al., 2006) and is strongly implicated in the control of cortical oscillations (Liu et al., 2015)—especially alpha rhythms (Omata et al., 2013). Thus, we sought to investigate whether neurocognitive modulation of cortical oscillations could impact dopamine transmission in the frontal cortex (FC), ACC, and thalamus. Specifically, we examined whether directly manipulating the dominant EEG oscillation, the alpha rhythm, may be associated with an endogenous release of dopamine using *in vivo* positron emission tomography (PET) imaging. An innovative way that alpha rhythms can be modified is with neurofeedback (NFB), a technique that enables users to control their brain activity using a closed-loop feedback. We have ourselves conducted extensive validation of alpha-desynchronizing NFB, which involves suppressing alpha rhythms below their resting-state baseline levels. First and foremost, we have found that this NFB protocol can be quickly learned by naïve healthy participants (Ros et al., 2010, 2013) as well as psychiatric patients (Kluetsch et al., 2014), while demonstrating its robust neurobehavioral effects in the direct aftermath of NFB i.e., up to 30 min after termination of training. Our first study demonstrated that one session of alpha-desynchronizing NFB was able to induce plastic increases in cortical excitability and decrease intracortical inhibition by circa 150%. Although long-hypothesized (Lubar, 1997), no studies have yet examined whether NFB effects may be associated with changes in the brain’s neurochemical status.

Hence, through a combined PET and EEG experiment, our study investigated whether NFB induces an upregulation of dopamine transmission in key brain nuclei using the radiotracer [^18^F]Fallypride. Development of high-affinity radioligands for the D_2/3_ receptor such as [^18^F]Fallypride have enabled non-invasive assessment of extrastriatal D_2/3_ receptor densities during pharmacologic (Slifstein et al., 2010) and behavioral paradigms (Albrecht et al., 2014). For example, PET experiments with [^18^F]Fallypride in monkeys showed that amphetamine challenge may induce a striking reduction in binding in the anterior cingulate cortex (ACC) (Mukherjee et al., 1997). It has also been shown that a single [^18^F]Fallypride scan protocol and linearized simplified reference region modeling (LSSRM) analysis can be used to measure extrastriatal dopamine release induced by a behavioural task (Christian et al., 2006; Lataster et al., 2011). Given that dopamine is widely implicated in cognitive control and neural plasticity through neuromodulatory projections to several cortico-subcortical sites, investigating its anatomical release could provide important insights on the real value of neurofeedback approaches for brain disorders such as attention deficit hyperactivity disorder and schizophrenia. Our main hypotheses were that: (i) desynchronizing NFB would induce a statistically greater decrease in alpha power, as well as lead to an increase in endogenous dopamine release in frontal cortex, anterior cingulate cortex and thalamus compared to the EMG biofeedback group, and that (ii) dopamine release will be positively correlated to the degree of alpha desynchronization during NFB.

## Materials and Methods

### Study Design and Sample Size

This was a pilot, randomized, controlled study with two independent participant groups (healthy young adults, males and females, aged 20–40) sampled through the Geneva Neuroscience Center subject pool: (i) an experimental neurofeedback (NFB) group (*n* = 16; 26.1 ± 5.2 years old; nine males and seven females) and (ii) a control EEG-electromyography (EMG)-biofeedback group (*n* = 16; 25.5 ± 5.4 years old; nine males and seven females). Prior to the study, written informed consent was obtained from each participant. The study was approved by the Research Ethic Committee of the Republic and Canton of Geneva. We excluded participants with past or current psychiatric or neurological disorders, past or current clinically significant medical condition and central nervous system disorder, addictive disorders (except tobacco), or current psychotropic treatment.

### PET Imaging

**FIGURE 1:**
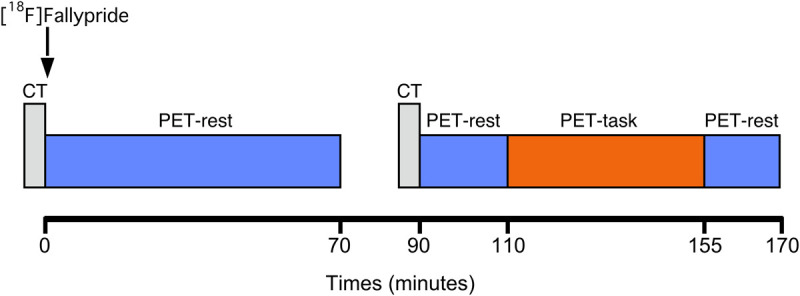
Experimental timeline of the study. PET-rest: eyes-open resting state; PET-task: eyes-open desynchronizing NFB (experimental group) or EMG biofeedback (control group).

All subjects were examined with PET using the D_2/3_ receptor antagonist radiotracer [^18^F]Fallypride. A custom-fitted thermoplastic mask was made for each participant and used to minimize head movement during the PET measurements. As depicted in Figure 1, a low dose (20 mA-s and 120 kV) computerized tomography (CT) scan of the head was acquired prior to the PET acquisition for attenuation correction of the PET data. Subjects then received a 10-s bolus i.v. injection of [^18^F]Fallypride at a specific radioactivity greater than 74 GBq/mmol Mean injected dose was 184.9 ± 14.9 MBq for the NFB group and 184.6 ± 10.1 MBq for the EMG group. A PET dynamic emission was initiated simultaneously on radiotracer injection and was acquired in 3-dimensional mode using a Biograph mCT Flow tomograph (Siemens medical solutions, United States, Inc.). The in-plane resolution of the scanner was approximately 4 mm full width at half-maximum. The PET emission scan was acquired in two dynamic scanning sequences, following a previously reported one-day PET protocols with modifications (Christian et al., 2006; Lataster et al., 2011). The first scanning sequence, with a duration of 70 min, represented baseline [^18^F]Fallypride kinetics, during which subjects lied down with their eyes open. Data were acquired in 60-s frames during the first 6 min and in 120-s frames thereafter. The baseline session was followed by a break period of 20 min, outside the scanner. After the break, subjects were repositioned on the PET scanner and a second low dose CT of the head was acquired immediately prior to the second dynamic PET scanning sequence for coregistration purpose to the first PET scanning sequence. A second emission dataset was then recorded for another 80 min (40 frames for 2 min/frame). In order to ensure that a possible displacement of radioligand induced by the task did not proceed from an “activation” due to the break, no task was presented during the first 20 min of this second emission scan.

At 110 min post-injection, the NFB or EMG-biofeedback task was initiated and performed for the 45 min, after which dynamic imaging continued in eyes open resting-state for another 15 min. EEG was simultaneously coregistered with PET (Kjaer et al., 2002), and attenuation correction was based on mu-map approximation of air/tissue boundary of the head. Additionally, on a separate day, a high-resolution T1-weighted and standard transverse T2-weighted brain magnetic resonance image (MRI; 1.5 Tesla; Signa; General Electric, Milwaukee, WI, United States) scan was performed in each subject for anatomical coregistration and to exclude structural brain abnormalities.

### EEG Recording

A multichannel EEG cap was used to measure whole-scalp activity simultaneously during the PET recording. Specifically EEG measurements were made for 3-min under eyes open conditions before and after, and for 45 min during each NFB/EMG session. The scalp signals were recorded using a 19 Ag/AgCl electrodes cap (Electro-cap International, Inc.^1^), according to the 10–20 international system. The ground electrode was placed on the scalp equidistant between Fpz and Fz. Electrical signals were amplified with the 21-channel Mitsar EEG system (Mitsar-201, CE0537, Mitsar, Ltd.^2^) and all electrode impedances was set to below 5 kOhm. For online recording, electrodes were referenced to linked earlobes, and then the common average reference was calculated off-line before further analysis. EEG data was continuously recorded at a sampling rate of 250 Hz, and then filtered with a off-line bandpass filter of 0.5–50 Hz.

### Neurofeedback and EMG-Biofeedback Procedure

Each NFB/EMG session lasted 45 min in total. The NFB session consisted of “alpha” amplitude desynchronization (i.e., down-regulation) at midline parietal cortex [for a detailed description see Ros et al. (2013)]. In brief, the Pz channel was specifically used for neurofeedback, using a Pro-Comp amplifier interfacing with the EEGer 4.2 neurofeedback software (EEG Spectrum Systems, CA, United States). Separate ground and reference electrodes were placed on the right and left earlobe, respectively. Pz was selected as the electrode overlying the posterior parietal cortex, whose metabolic changes have been previously linked to EEG alpha rhythm modulation. All participants interacted with a “SpaceRace” game where they received continuous visual feedback in the form of a moving spaceship and a dynamic bar graph whose height was inversely proportional to real-time alpha amplitude fluctuations. Participants were told that the spaceship would move forward whenever they were “in-the-zone” of their target brain activity (i.e., alpha lower than threshold), and that it would stop when they were “out-of-the-zone” (i.e., alpha higher than threshold). The aim of the training was to use the feedback they received during the game to learn to keep the spaceship traveling through space. For the purpose of online NFB training, the EEG signal was infinite impulse response band-pass filtered to extract alpha (8–12 Hz) with an epoch size of 0.5 s. Participants were rewarded upon suppression of their absolute alpha amplitude. For each participant, the reward threshold was initially set so that their alpha amplitude would fluctuate below the initial 3-min baseline average approximately 60% of the time (i.e., they received negative feedback about 40% of the time). To ensure that all participants received comparable frequencies of reward, we readjusted their reward thresholds to meet the desired ratio, when they achieved disproportionately higher (>80%) or lower (<40%) rates of reward during feedback. The entire NFB session was divided into 15 × 3 min training periods with a short break (1 s) after each period. During the breaks, the scores for the preceding periods were displayed.

Electromyographic (EMG) biofeedback (Degood and Chisholm, 1977) was presented using the same feedback interface and reward parameters as for NFB [i.e., 15 × 3 min training periods with a short break (10 s) after each period]. This was based on downregulating (relaxing) the EMG power (20–45 Hz) from the facial jaw muscle with an electrode on the right masseter muscle. This condition was used to control for visual stimuli exposure (same visual feedback game as NFB) and feedback-related cognitive control.

### PET Data Analysis

Reconstructed SPECT images were processed using the PMOD V3.9 software (PMOD Technologies Ltd., Zurich, Switzerland). First, the second PET scanning sequence was co-registered to the first one using their respective CT scans. Both sequences were then merged to create a single dynamic PET sequence. To minimize the effects of head movement, PET images underwent frame-to-frame realignment and were coregistered to individual T1-weighted MRI. Regions of interest (ROI) for the thalamus, frontal cortex, anterior cingulate cortex, and cerebellum were drawn on the MRI and applied to the dynamic PET images to produce time-activity curves (TACs). Non-linear least squares fitting analyses based on the linear extension of the simplified reference region model (LSSRM; Alpert et al., 2003), using the cerebellum as a reference, were applied to the 170 min of [^18^F]Fallypride TAC data, to estimate the non-displaceable binding potential (BP_ND_) as an index of D_2/3_R availability, and γ as an index of AMPH-induced DA release in the thalamus, frontal and anterior cingulate cortices. In brief, the LSSRM takes into account temporal perturbations in radioligand specific binding caused by pharmacological or non-pharmacological-induced changes in endogenous levels of neurotransmitter such as dopamine during a single-scan session (Alpert et al., 2003). The LSSRM assumes that a steady physiological state is disturbed at a certain time of the experiment and allows the dissociation rate of the radioligand from the receptor, k_2a_, to change over time in response to local variation in dopamine concentration [*k*_2a_ = k_2_/(1 + BP_ND_)], where *k*_2_ is the tissue-to-plasma efflux constant in the target region. Changes in BP_ND_ in competition studies are assumed to reflect inverse variations in the concentration of extracellular neurotransmitter (Ginovart, 2005). Competition between dopamine and radioligand for binding on receptors is reflected by a temporal change of *k*_2a_, which is accounted for by a time-dependent parameter *k*_2a_ + γ ⋅ h(t), where γ represents the amplitude of the radioligand displacement and the function h(t) describes a rapid change following competition onset and dissipation over time. The decay function h(t) = exp[−τ(t−T)] denotes temporal fluctuation in the model parameters, where τ controls the rate at which competition effects die away and T represents the time of competition onset. Therefore, an increased in *k*_2a_, reflected by a decrease in BP_ND_ caused by an increased in task-induced dopamine release results in a positive value of γ. Here, T was set to the time of NFB initiation (ie., 110 min post-radiotracer injection), and τ was set to 0.03 min^–1^ in accordance with previous investigations of behavioral interventions with [^18^F]Fallypride (Christian et al., 2006; Lataster et al., 2011; Ceccarini et al., 2012; Kasanova et al., 2018). The entire set of time-activity data (170 min) was included in the LSSRM fitting procedure.

### EEG Data Analyses

These were conducted with a combination of EEGLAB^3^ and the Neurophysiological Biomarker Toolbox^4^ in Matlab. For offline analyses, EEG signals were re-referenced to common-average reference. Low- and high-pass filters were set to 0.5 and 40 Hz, respectively, with a 55–65 Hz notch filter. We used ICA decomposition to first remove stereotypical artifacts using the Infomax algorithm (blinking and lateral eye movement). Statistically defined artifacting was then carried out with the FASTER plug-in (Nolan et al., 2010) removing segments based on extremal deviations of amplitude and variance from the mean. Then, resting-state EEG power was calculated offline using the Short Time Fourier Transform (STFT) in 4-s epochs (50% overlapping with Hanning window) in each of the following bandwidths: delta (1–4 Hz), theta (4–8 Hz), alpha (8–12 Hz), and beta (13–25 Hz). Higher frequencies (gamma > 25 Hz) were not analyzed as they may easily be contaminated by muscle artifact throughout the extended NFB session. Given the low anatomical specificity of EEG and the hypothesis of a generalized effect on neurotransmission, all analyses were conducted on the mean of all 19 EEG channels. The normalized training EEG change for each participant was estimated by the ratio of the average EEG amplitude across the whole biofeedback training period and the first baseline EEG, and designated as “training EEG change.” Likewise, the normalized change in the baseline EEG amplitude was expressed by the ratio of the second divided by the first baseline, and designated as “resting EEG change.”

### Statistical Analysis

Between-group differences in [^18^F]Fallypride BP_ND_, γ, and *t*-scores were analyzed using a two-way ANOVA, with the brain region as the within-subject factor and the treatment group (EMG or NFB) as the between-subject factor.

The *t*-scores derived for γ based on the covariance matrix of the parameter as estimated by the LSSRM fitting procedure were used to assess the statistical significance of task-induced dopamine release (Christian et al., 2006). According to the model, the *t*-scores (*t* = γ/SD(γ), where SD(γ) is the standard error parametric value for (γ) represent effect sizes for DA release during the task. With a degree of freedom of 75, a threshold of *t* > 2.4 was used to represent *P* < 0.05 with a one-tailed *t*-test (Christian et al., 2006).

To test for group/condition differences in *EEG absolute power spectrum*, we used a permutation test with 5,000 repetitions on all channels, and subsequently corrected for multiple comparisons using binomial correction. The significance threshold for all comparisons was set to alpha = 0.05.

The quantitative relationship between dopamine release and EEG change was investigated using a Pearson correlation analysis, where intra-individual alpha changes (training EEG change, resting EEG change) were used as predictors of task-induced dopamine release (γ).

## Results

### PET Signatures During NFB and EMG Training

[^18^F]Fallypride BP_ND_ values obtained in the EMG- and NFB-treated subjects are shown in Table 1. A two-way ANOVA for BP_ND_ with both region and treatment group as main factors revealed a significant main effect of region (*F*_2, 90_ = 1,167; *p* < 0.001) but no main effect of treatment (*F*_1, 90_ = 3.2; *p* > 0.05) and no interaction between region and treatment (*F*_2, 90_ = 0.35; *p* > 0.05). BP_ND_ values between the NFB and EMG groups were not statistically different in any brain regions investigated, indicating the two groups were physiologically well-matched at baseline. BP_ND_ values of circa 0.60 were obtained in the ACC and in the FC for both treatment groups, and were 5–6 and 7–8 times lower, as expected, that those obtained in the thalamus where dopamine receptor binding is known to be stronger.

**TABLE 1:** [^18^F]Fallypride BP_ND_ values obtained in the NFB and EMG treatment groups.

	**NFB**	**EMG**
Anterior cingulate cortex	0.62 ± 0.13	0.55 ± 0.11
Frontal cortex	0.47 ± 0.12	0.38 ± 0.11
Thalamus	3.33 ± 0.47	3.14 ± 0.34

*Values are means ± SD.*

**FIGURE 2:**
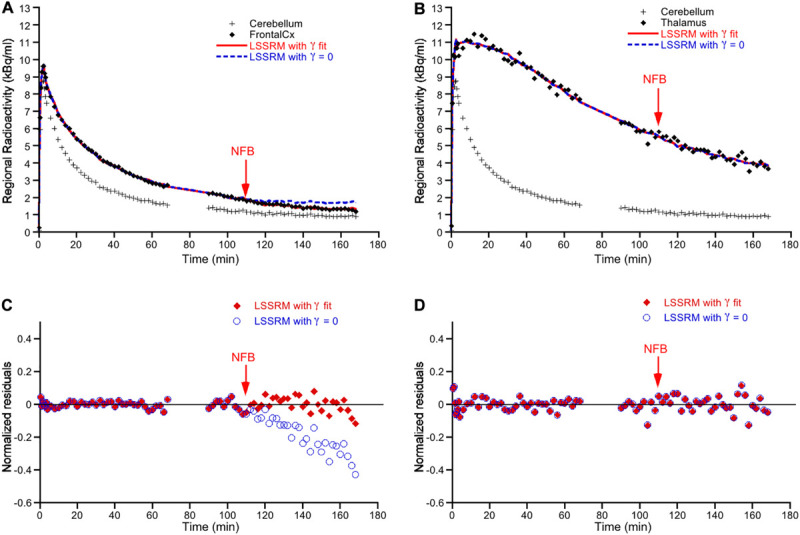
Representative time-activity curves of [^18^F]Fallypride binding in the frontal cortex **(A)** and thalamus **(B)** in one subject. Time-activity data in cerebellum, used as reference region in the model, is also displayed. The vertical arrow represents the time at which neurofeedback (NFB) was initiated (i.e., 110 min post-radiotracer injection). For each target brain region, the symbols correspond to the experimental measured values, the solid red line corresponds to the fitted curve obtained according to the LSSRM with γ fit, and the dashed blue line corresponds to the fitted curve obtained according to LSSRM but with γ fixed to 0. The LSSRM with γ fit yielded a *t*-score of 9.89 in frontal cortex and 0 in thalamus in this subject. Panels **(C,D)** show the normalized residuals [(PET – model)/PET] of the model fit with the γ parameter (closed red symbols) and with γ fixed to zero (blue open symbols) in frontal cortex and thalamus, respectively. The inclusion of γ in the LSSRM led to an improvement of the model fit in frontal cortex but not in thalamus.

**FIGURE 3:**
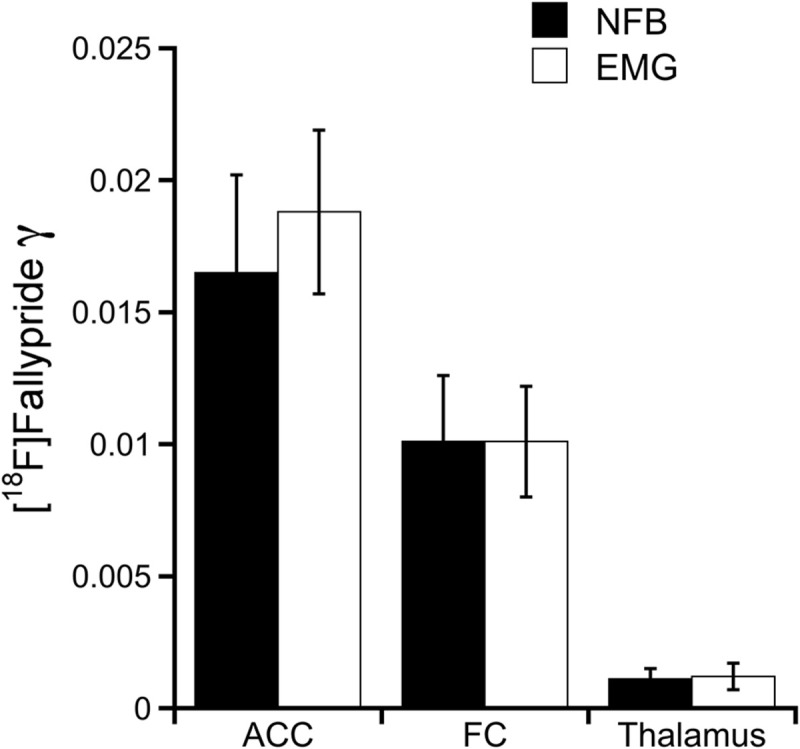
Endogenous dopamine release during NFB and EMG conditions. Bar graphs with NFB and EMG group γ parameter values {i.e., amplitude of [^18^F]Fallypride ligand displacement} for hypothesized regions-of-interest: anterior cingulate cortex (ACC), frontal cortex (FC), and thalamus. No statistically significant differences in were detected between groups.

Figure 2 displays representative examples of [^18^F]Fallypride time-activity curves obtained in the FC (Figure 2A) and the thalamus (Figure 2B) in one subject treated with NFB. When including γ in the model, the LSSRM fits indicated a decrease in [^18^F]Fallypride binding promptly after the initiation of NFB in FC but not in thalamus, indicating a rapid task-induced release of DA in the former but not in the latter brain region (Figure 2A). Plots of the normalized residuals for the LSSRM fits to the TAC data with and without the γ parameter are shown in Figures 2C,D for the FC and thalamus, respectively. The inclusion of γ in the LSSRM led to an improvement of the model fit in FC but not in thalamus. The γ estimates obtained in the NFB and EMG treatment groups are shown in Figure 3. A significant main effect of brain region (*F*_2, 90_ = 24.1; *p* < 0.001) but no effect of treatment (*F*_1, 90_ = 0.17; *p* > 0.05) or treatment × brain region interaction (*F*_2, 90_ = 0.15; *p* > 0.05) was found on γ, indicating that task-induced dopamine release differed between brain regions but not between tasks. In the FC and ACC, all subjects yielded a positive γ parameter during NFB, with mean *t*-scores of 8.9 and 8.3 in the two regions, respectively. In the thalamus, there was no case of a significant γ parameter with a mean *t*-score of 1.2, suggesting no NFB-induced dopamine release in this region. The mean *t*-scores for γ estimated for EMG-induced dopamine release were 10.7, 9.6, and 0.98 in the ACC, FC, and thalamus, respectively. A significant main effect of brain region (*F*_2, 90_ = 29.5; *p* < 0.001) but no effect of treatment (*F*_1, 90_ = 0.92; *p* > 0.05) or treatment × brain region interaction (*F*_2, 90_ = 0.36; *p* > 0.05) was found on *t*-scores, further indicating that task-induced DA release differed between brain regions but not between treatment.

### EEG Signatures During NFB and EMG Training

At baseline, no significant differences (*p* < 0.05) were detected between NFB and EMG groups in global absolute power for delta, theta, alpha, or beta bands.

**FIGURE 4:**
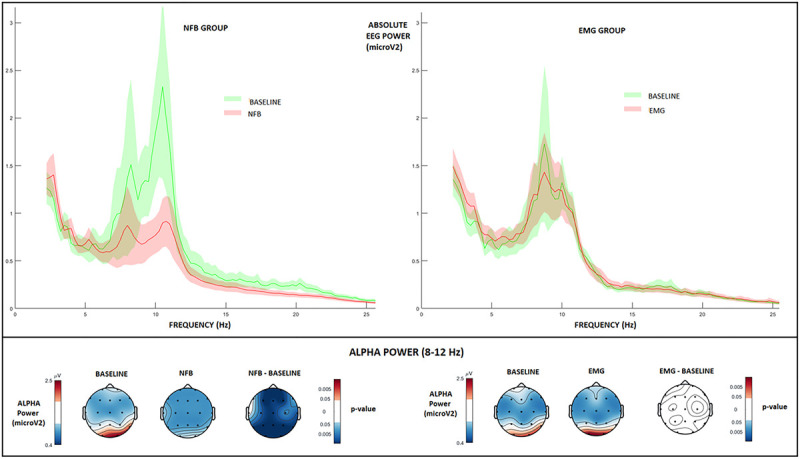
EEG absolute power changes during NFB and EMG conditions. Top: EEG absolute power spectrum during baseline (green), and NFB/EMG (red) in NFB (left) and EMG (right) groups. Solid lines: mean value at the parietal (Pz) feedback electrode, highlighted areas: standard error interval. Bottom, first row: Topographic plots of absolute alpha amplitude during baseline and NFB, and paired permutation test *p*-values (binomial corrected, *p* < 0.05). Bottom, second row: Topographic plots of absolute alpha amplitude during baseline and EMG, and paired permutation test *p*-values (binomial corrected, *p* < 0.05).

As can be seen from Figure 4 (left panel), channel-wise permutation tests indicated that alpha power was significantly reduced during NFB as compared to resting-state baseline (NFB – baseline, binomial corrected, and *p* < 0.05), demonstrating that participants successfully downregulated their alpha amplitude in the direction of the neurofeedback protocol. This is in line with several earlier NFB studies demonstrating a similar alpha-desynchronization effect in healthy subjects (Ros et al., 2010, 2013).

On the other hand, Figure 4 (right panel) indicates that the EMG group did not significantly alter their resting-baseline alpha power during EMG biofeedback (EMG – baseline, binomial corrected, n.s.). This can probably be attributed to the more relaxation-inducing nature of EMG biofeedback, which is based on reducing muscle tension without inducing alpha desynchronization (Degood and Chisholm, 1977).

### Associations Between Dopamine Release and EEG

**FIGURE 5:**
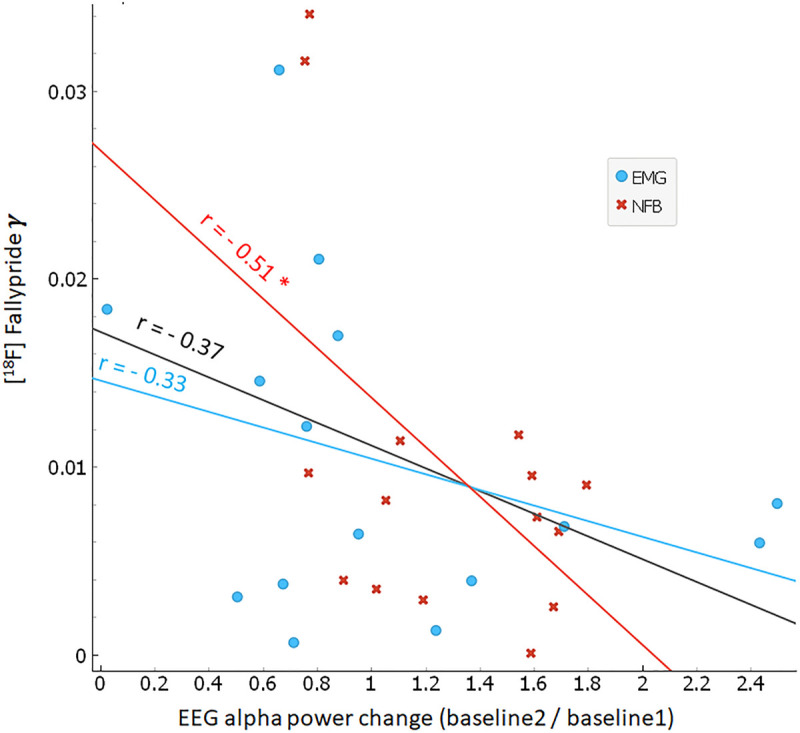
Correlation between endogenous dopamine release and baseline alpha power change. Scatter plot between frontal cortex dopamine release (γ; y-axis) and baseline change in alpha power (ratio of *baseline2*/*baseline1*; x-axis). NFB group subjects are indicated by red crosses, EMG group subjects by blue circles. Red, blue, and black lines indicate linear regression fits for NFB (*r* = –0.51, *p* < 0.05), EMG (*r* = –0.37, n.s.), and pooled NFB + EMG groups (*r* = –0.33, n.s.), respectively.

Correlation analyses between inter-individual differences in alpha power during NFB (i.e., training EEG change) and task-induced DA release (i.e., γ) did not reveal significant associations for any brain region (n.s.). Hence, we did not confirm our secondary hypothesis that the degree of alpha desynchronization during NFB would significantly predict dopamine release. However, exploratory analyses examining the relationship between task-induced DA release (i.e., γ) and *pre-to-post* task changes in baseline alpha power (i.e., resting EEG change) revealed a significant association. As can be seen in Figure 5, there was a negative correlation between γ in the FC and inter-individual changes in baseline alpha power: *r* = −0.51 for the NFB group (*p* < 0.05), *r* = −0.33 for the EMG group (n.s.), and *r* = −0.37 for the pooled (NFB + EMG) data (n.s.). This suggests that greater levels of dopamine release during NFB resulted in larger decreases of spontaneous alpha power *pre-to-post* NFB.

## Discussion

The aim of the present study was to gain a better understanding of the neurochemical effects of NFB on the brain. To our knowledge, this is the first NFB-PET application and attempt to detect endogenous dopamine release through an alpha-desynchronization protocol using [^18^F]Fallypride. Our study revealed that both NFB and EMG caused a significant, but comparable, decrease in [^18^F]Fallypride binding in the anterior cingulate and frontal cortical regions, an effect that indicate, albeit indirectly, task-related dopamine release in these brain regions. However, and contrary to our hypothesis, no task-related increase in dopamine release was observed in thalamus. In addition, the reduction in EEG alpha power during NFB did not significantly correlate with dopamine release in any brain regions, suggesting that alpha-desynchronization *per se* does not directly influence dopamine release. However, we did observe correlational evidence for the reverse relationship, whereby the degree of dopamine release in frontal cortex significantly predicted decreases in spontaneous alpha power *pre-to-post* NFB. This is an interesting finding, as it supports a delayed (rather than instantaneous) effect of dopamine release on an EEG functional brain measure such as alpha power. Moreover, the observed negative relationship between alpha power and dopamine release is consistent with a previous EEG-PET study in humans (Kjaer et al., 2002), suggesting that intra-individual changes in alpha power post-NFB could be partly associated with changes in dopamine tone.

Because of its high affinity and long half-life, [^18^F]Fallypride offers the possibility to explore D_2/3_ receptor-mediated signaling in both striatal and extrastriatal regions (Mukherjee et al., 1999, 2002). However, due to the high concentration of D_2/3_ receptors in striatum, [^18^F]Fallypride binding kinetics are relatively slow in this brain region and scan durations of 180 min are needed to reliably reach equilibrium and achieve stable BP_ND_ in striatum (Christian et al., 2000; Vernaleken et al., 2011). In extrastriatal regions such as the cortex and thalamus, where D_2/3_ receptor densities are one to two orders of magnitude lower than in the striatum (Kessler et al., 1993), equilibrium of [^18^F]Fallypride binding is reliably reached within 60 min of scan duration (Vernaleken et al., 2011). In the present study, task timing, which is thus critical for assessing striatal and extrastriatal dopamine release, was chosen and optimized for extrastriatal regions and did not permit a concomitant evaluation of striatal dopamine release. Consistent with previous studies (Lataster et al., 2011, 2014; Ceccarini et al., 2012; Vrieze et al., 2013; Hernaus et al., 2015), we found that [^18^F]Fallypride and the LSSRM single scan approach can be successfully used to detect dopamine released in cortical regions during task performance. Moreover, the BP_ND_ values estimated in the frontal and cingulate regions were consistent with those reported in previous studies (Mukherjee et al., 2002; Cropley et al., 2008).

During the performance of both NFB and EMG, increases in dopamine release were observed in the ACC and FC. The implications from these findings are interesting in relation to cortical regions and their neurotransmitter response to an active task. As there was an effect observed regardless of treatment group, this indicates that both tasks similarly induced dopamine release but that this effect was preferentially circumscribed to cortical regions as it was not observed in subcortical regions such as thalamus. Interestingly, converging evidence suggest that dopamine signaling in the PFC is essential for motivation and for promoting attention during goal-directed behaviors (Bilder et al., 2004; Costa, 2007; Assadi et al., 2009). On the other hand, the ACC has been proposed to play a central role in using action outcomes to guide future behaviors and to be involved in the processing of negative feedback information (Williams et al., 2004; Hayden and Platt, 2010). Dopamine release in both groups may have occurred as a result of the positive reward and/or negative feedback presented to subjects during neurofeedback gameplay. This is a plausible assumption, insofar other studies have found that reward-correlated information is encoded in low-frequency signals (<32 Hz) within the dopaminergic system (Pasquereau et al., 2019). Moreover, since neurofeedback is based on closed-loop feedback, it has been proposed this involves error-prediction (Ros et al., 2014), which has historically been linked to dopamine signaling (Schultz et al., 1997). Nevertheless, both goal-directed tasks and video games have been found to be linked to dopamine release, without any intent to control cortical oscillations (Koepp et al., 1998; Vrieze et al., 2013; Kasanova et al., 2018). The present work raises questions to be addressed by future studies with regard to the experimental protocol between treatment groups. Specifically, the goal of examining dopamine release induced by an alpha-desynchronization neurofeedback protocol may not be optimal, given that the visual neurofeedback task conditions do not control for the potential influence of a goal-directed task or a video-game type interface.

On the other hand, we regard this a pilot “proof-of-concept” study and future refinement may be necessary. In addition to ROI analysis, previous studies investigating task-induced dopamine release using the LSSRM have used voxel-based parametric images of *t*-scores to quantify the spatial extent of task-activated voxels exceeding a significant *t*-score threshold (Christian et al., 2006). Indeed, task-induced activations are not necessarily associated with a sharp peak of dopamine release, and can also manifest as spatially distributed dopamine activation events within certain brain areas. Such a spatial-extent-based approach is generally viewed as more sensitive (Poline et al., 1997), and has been successfully used to detect the spatial extent of task-induced dopamine neuromodulation (Christian et al., 2006; Lataster et al., 2011; Ceccarini et al., 2012; Kasanova et al., 2017). Large ROIs contain a large number of voxels and taking the mean of all the voxels in one ROI may lessen the significance of the small population of voxels that may have been exhibiting activation (Davis et al., 2014; Tong et al., 2016). Using voxel-wise parametric maps rather than ROI-based analysis to measure changes in D_2/3_ receptor binding is deemed a better way to deal with inter-subject variability that may be induced by the decreasing accuracy of activation measures caused by the larger radius of an entire region of interest (Oosterhof et al., 2011; Matheson et al., 2017). Voxel-by voxel analyses would allow to preserve spatial resolution, improve signal-to-noise ratio, and provide high-quality binding parametric images and reliable and regionally specific parameter estimates (Friston et al., 1994; Tomasi et al., 2009; Odano et al., 2017). It would enable refinement of sub-regions in already highlighted regions of interest, where activation may have been masked within a region, as voxel-by-voxel analysis is more sensitive to fluctuations compared to ROI analysis. Put simply, minimal visual inspection of parametric maps may be better at detecting phenomena invisible to ROI analysis, such as a task-associated effects in smaller regions/subdivisions of the brain (Tomasi et al., 2009).

## Conclusion

Our aim was to assess the effect of EEG-based neurofeedback on endogenous dopamine release using PET imaging. By use of the radiotracer [^18^F]Fallypride, we were able to measure D_2/3_ receptor activity in target brain regions, and by applying a linearized version of the simplified reference tissue model (LSRTM), we were able to quantify ligand displacement and receptor density. It was hypothesized that there would be a statistically significant increase in endogenous dopamine release in the neurofeedback group in the FC, the ACC, and in the thalamus, and that a differential effect would be observed in the EMG group. Our observations showed that, contrary to our hypothesis, both NFB and EMG treatment induced similar increases in dopamine release and that this effect was restricted to cortical regions. We are thus unable to conclude that neurofeedback differentially induces endogenous dopamine release, and further investigations in this area are suggested to gain a deeper understanding of neurofeedback’s potency in inducing dopamine release, and its specific ability to alter neuromodulatory pathways. Future replication of our work is warranted using different stimuli between experimental groups in order to better distinguish the effect of neurofeedback on dopamine release. We hope that this investigation will lead to further studies on neurofeedback’s prospective ability to induce measurable changes in brain function and brain plasticity.

## Data Availability Statement

The raw data supporting the conclusions of this article will be made available by the authors, without undue reservation.

## Ethics Statement

The studies involving human participants were reviewed and approved by Commission Cantonale d’Ethique de la Recherche (CCER). The patients/participants provided their written informed consent to participate in this study.

## Author Contributions

TR and NG contributed to the study conception, study design, study supervision, data analysis, data interpretation, manuscript drafting, and manuscript revision. TR and VG also contributed to the data collection. PV and VG contributed to manuscript drafting and revision. JK, TA, and AM contributed to the data analysis and statistical analysis. All authors contributed to the article and approved the submitted version.

## Conflict of Interest

The authors declare that the research was conducted in the absence of any commercial or financial relationships that could be construed as a potential conflict of interest.

## Footnotes

1 www.electro-cap.com

2 http://www.mitsar-medical.com

3 http://sccn.ucsd.edu/eeglab/

4 http://www.nbtwiki.net/

## Abbreviations

PET: positron emission tomography
EMG: electromyography
EEG: electroencephalography.
